# Effects of Mortality Salience on Physiological Arousal

**DOI:** 10.3389/fpsyg.2019.01893

**Published:** 2019-08-20

**Authors:** Johannes Klackl, Eva Jonas

**Affiliations:** Department of Psychology, University of Salzburg, Salzburg, Austria

**Keywords:** mortality salience, physiological arousal, arousal misattribution, threat and defense, worldview defense

## Abstract

Making the inevitability of mortality salient makes people more defensive about their self-esteem and worldviews. Theoretical arguments and empirical evidence point to a mediating role of arousal in this defensive process, but evidence from physiological measurement studies is scarce and inconclusive. The present study seeks to draw a comprehensive picture of how physiological arousal develops over time in the mortality salience (MS) paradigm, and whether contemplating one’s mortality actually elicits more physiological arousal than reflecting on a death-unrelated aversive control topic. In a between-subjects design, participants were asked two open questions about their mortality or about dental pain. Cardiac, respiratory, and electrodermal indicators of arousal were measured both as participants provided written answers to the questions, and during a series of resting intervals surrounding the questions. A Bayes factor analysis indicated support for the hypothesis that the MS paradigm increases physiological arousal, both while answering the two open-ended questions and afterward. Regarding the MS versus dental pain comparison, the null hypothesis of no difference was supported for most analysis segments and signals. The results indicate that the arousal elicited by MS is not different from that elicited by dental pain salience. This speaks against the idea that worldview defense following MS occurs because MS produces higher physiological arousal. Of course, this finding does not rule the importance of other forms of arousal (i.e., subjective arousal) for MS effects.

## Introduction

A considerable number of studies have shown that using reappraisal and misattribution techniques aimed at reducing arousal eliminates defensiveness in response to reminders of one’s mortality (Greenberg et al., 2003; Webber et al., 2015), and other psychological threats (Ben-Zeev et al., 2005; Proulx and Heine, 2008; Kay et al., 2010; Nash et al., 2011; Greenaway et al., 2015). This suggests that arousal plays a key role for the emergence of defense in response to threat, including mortality threat. However, few of these studies have actually measured physiological arousal. The goal of the present study is to fill this gap and provide a detailed investigation of how physiological arousal develops in the most commonly used threat induction paradigm: the mortality salience paradigm.

Mortality salience (MS) is typically evoked by asking participants two open-ended questions about their death. MS is typically manipulated between subjects, with the control group receiving similar open-ended questions about a death-unrelated topic, most commonly dental pain. Hundreds of published studies suggest that relative to reflecting on death-unrelated topics, reflecting on mortality makes people defensive about their worldviews and their self-esteem, and increases their commitment to close relationships (Burke et al., 2010).

Terror management theory (TMT) argues that reminders of mortality increase the potential for experiencing existential anxiety, and that this potential is kept in check by a dual-component anxiety buffer consisting of worldviews and self-esteem (Greenberg et al., 1986). While worldviews convey standards about how one ought to live, self-esteem reflects individual beliefs about the extent to which one is living up to the standards set by those cultural worldviews. The MS hypothesis, which is derived from TMT, states that to the extent that a psychological structure provides protection against anxiety, reminding people of the source of their anxiety should lead to an increased need for that structure and, thus, more positive reactions to things that support it and more negative reactions to things that threaten it. In a nutshell, according to TMT awareness of life’s finitude temporarily raises the potential for anxiety whereas bolstering worldviews and self-esteem lowers it. Close relationships seem to constitute a third buffer against mortality concerns in addition to worldviews and self-esteem (Florian and Mikulincer, 1998; Mikulincer and Florian, 2000; Mikulincer et al., 2003).

Although the effectiveness of the MS manipulation itself is well supported by empirical evidence, the psychological processes mediating its effects are unclear. The most elegant test of TMT’s original proposition that a heightened potential for anxiety underlies MS effects is provided by one study which found that consuming an anxiety-blocking placebo (thereby eliminating the potential for anxiety) prevents heightened worldview defense after MS (Greenberg et al., 2003). According to Terror management scholars, the potential for anxiety can also be inferred using measures of death-though accessibility (DTA) (Pyszczynski et al., 1999; Greenberg et al., 2003). DTA is commonly measured using word fragment completion tasks. For example, COFF_ can be completed either to form the death-related word COFFIN, or the death-unrelated word COFFEE. DTA is inferred from the number of fragments completed in a death-related manner (Hayes et al., 2010). MS does seem to increase DTA (Greenberg et al., 1994; Arndt et al., 1997; Trafimow and Hughes, 2012; Echebarria Echabe and Perez, 2016), and DTA has also been shown to mediate MS effects (Echebarria-Echabe, 2013; Echebarria Echabe and Perez, 2016). However, there is a methodological problem with DTA: measuring DTA may inadvertently increase the salience of death thoughts (Hayes and Schimel, 2018), thereby affecting the purported mediating process.

Apart from DTA, negative affect and self-esteem have also been discussed as mediators of MS effects. Although terror management scholars have claimed that mortality salience does not lead to reliable increases in negative affect (Simon et al., 1997; Greenberg et al., 2001), and that it is the potential for anxiety rather than anxiety itself that is responsible for MS effects to occur, studies employing more specific measures have revealed that, in comparison to neutral topics, mortality salience actually elicits negative affective states including fear, anxiety, sadness, and sorrow (Kastenbaum and Heflick, 2010; Echebarria-Echabe, 2013; Lambert et al., 2014; Echebarria Echabe and Perez, 2016). Because affective states can bias evaluative judgments (Schwarz and Clore, 1983, 2003; Schwarz, 2012), it seems plausible that these affective changes are partly responsible for the exaggerated evaluative judgments on worldview- and self-esteem-relevant targets that are typically found as a consequence of mortality salience (Tritt et al., 2012). Some studies have indeed reported evidence for mediation of MS effects via affect (Echebarria-Echabe, 2013; Echebarria Echabe and Perez, 2016) even beyond commonly investigated outcomes like self-esteem, worldviews, and relationships (e.g., sexualized stimuli; Lee et al., 2017). Self-esteem modulations (Harmon-Jones et al., 1997; Lambert et al., 2014; Echebarria Echabe and Perez, 2016), although typically considered a “buffer” against existential anxiety in the framework of TMT (Schmeichel et al., 2009), has also been found to mediate MS effects (Echebarria Echabe and Perez, 2016). Finally, a study by Lambert et al. (2014) found that MS induces both negative affective and self-esteem bolstering tendencies that are somewhat antagonistic and cancel each other out. This suggests that negative affect and self-esteem are different, yet related mediators that operate in parallel.

Although MS manipulations do seem to enhance DTA, negative affect and self-esteem, these phenomena only mediate the effects of some (but not all) MS manipulations on some (but not all) kinds of defense (Echebarria Echabe and Perez, 2016). For example, the extent to which people felt worried after reflecting about death-related topics mediated the effect of MS on how much they endorsed xenophobic beliefs and intended to have a family and children; increases in DTA mediated the effect of MS on afterlife beliefs; and self-esteem mediated the effect of MS on how much participants identified with Europe (Echebarria Echabe and Perez, 2016). In other words, different aspects of peoples’ worldviews seem to be activated depending on how mortality is induced, and the mediating mechanisms also seem to vary (Echebarria-Echabe, 2013).

Another possible mediator of MS effects is arousal. Tritt et al. (2012) have proposed that mortality salience may lead to affective states that may not be accessible to verbal self-reports, but nevertheless manifest themselves in physiological arousal. Similarly, the meaning maintenance model (Proulx et al., 2012) claims that the awareness of mortality (and other threats) evokes aversive arousal, and that worldview defense represents a compensatory response aimed at reducing this arousal. Arousal is also central to alternative, general theoretical perspectives on mortality salience and threat and defense in general (Holbrook et al., 2011; Hart, 2014; Jonas et al., 2014). For the sake of brevity, we will refer to the claim that arousal underlies MS effects as the arousal hypothesis throughout this paper.

Studies using “cognitive” manipulations of arousal support the arousal hypothesis. For example, consuming a placebo that allegedly blocks anxiety has been found to eliminate the effect of mortality salience on worldview defense (Greenberg et al., 2003)., although it may also be counted as evidence for the idea that the potential for anxiety me. Arousal also seems to play a role in the context of other threats. Using reappraisal to downregulate arousal and attributing arousal to a neutral source have been shown to eliminate defensiveness in response to worldview threats (Webber et al., 2015), perceptual anomalies (Proulx and Heine, 2008), goal impedance (Nash et al., 2011), randomness (Kay et al., 2010), stereotype threat (Ben-Zeev et al., 2005) and low control (Greenaway et al., 2015). However, none of these studies have actually measured physiological arousal, although this would certainly provide an interesting piece of the puzzle.

The validity of the arousal hypothesis should not be judged solely on the basis of evidence from “cognitive” manipulations of arousal. This is because different physiological measures of arousal (including heart rate, respiration, or skin resistance) are weakly correlated, and self-reported arousal is only weakly correlated with physiological arousal. This problem seriously questions the construct validity of arousal itself (Clements et al., 1976; Revelle and Loftus, 1990). In fact, psychophysiologists have repeatedly emphasized that arousal or physiological activation is not a unidimensional construct, and that there is much independent variation of arousal in different physiological systems (Reisenzein, 1983; Heilman, 2000; Mauss et al., 2005). In light of these findings, it is not only interesting to consider a multitude of physiological arousal indicators when working with the concept of arousal; it is imperative. In addition, there is direct evidence that physiological reactivity can in fact mediate the relationship between anxiety and political attitudes (Renshon et al., 2015), although the latter study did not focus on death-related anxiety.

The few published studies on physiological arousal in the MS paradigm have revealed mixed results, which may be due to methodological differences. An early study sampled physiological arousal in the minute after participants filled out a questionnaire about their mortality, eating, or no questionnaire (Rosenblatt et al., 1989, Study 5). The study found no differences between these three conditions, but did not report whether engaging in these procedures elevated arousal over baseline levels. A recent virtual reality study indicated that taking a simulated walk in a graveyard (a reminder of mortality) increases low-frequency heart rate variability relative to taking a simulated walk in a public park (Chittaro et al., 2017). The disparity between these results may have various reasons, but one possibility is timing. Whereas in the Rosenblatt study, arousal was measured only after the manipulation, the Chittaro study only measured arousal during the manipulation. An additional problem is that neither study recorded arousal long after the mortality salience manipulation. This may be important, because effects of mortality salience on worldview defense do not emerge unless a delay of several minutes is introduced between the mortality salience induction and the worldview defense measurement (Pyszczynski et al., 1999). A similar delayed increase may be apparent for arousal. Together, existing physiological studies on mortality salience do not provide a comprehensive picture of how arousal develops in the MS paradigm because of narrow and arbitrarily placed sampling periods.

Another problem in arousal research that is also relevant to this investigation is that common measures of arousal, such as heart rate or electrodermal activity, are blind to whether arousal is positive or negative. The biopsychosocial model (Tomaka et al., 1993; Blascovich and Tomaka, 1996; Blascovich and Mendes, 2000) differentiates between challenge and threat types of cardiovascular arousal. Whereas both are associated with increases in heart rate, blood pressure, and reductions in pre-ejection period (PEP), challenge (i.e., when individuals think that personal resources exceed situational demands) leads to increased cardiac output (i.e., the amount of blood pumped in a given time) and decreased total peripheral resistance (a proxy for net constriction versus dilation of the arterial system). Under threat (i.e., when individuals think that situational demands exceed personal resources), the pattern is reversed, indicating more constricted blood vessels, and less blood pumped by the heart. For example, interacting with upward comparison partners (Mendes et al., 2001) and expectancy-violating partners (e.g., Asians with southern accents) (Mendes et al., 2007) leads to a cardiovascular threat pattern. To determine whether mortality salience evokes a cardiovascular threat or challenge pattern, we measured cardiac output and total peripheral resistance in addition to the more common measures of arousal.

The present study seeks to draw a more fine-grained picture of how arousal develops over time in the mortality salience paradigm, and whether mortality salience generates more physiological arousal than an aversive control condition (in this case, dental pain). We used a broad array of electrodermal, respiratory, and cardiac measures of arousal. We measured arousal before, during, and several minutes after reflecting on mortality (or dental pain, a frequently used aversive control topic). This study provides the most comprehensive investigation of physiological arousal in the mortality salience paradigm to date.

## Materials and Methods

### Participants

One hundred and fifteen participants (mean age = 22.93 years, *SD* = 3.21; 76 female, 39 male) took part in the experiment. Exclusion criteria, assessed by self-report, were current use of medication affecting the physiological systems under study (e.g., beta blockers). The study was approved by the ethics committee of the University of Salzburg. All participants signed informed consent forms, and could withdraw participation at any point, although no participant made use of this option. Physiological data from four participants were entirely lost because the recording computer crashed near the end of the experiment. Three additional physiological datasets were lost because the experimenter forgot to hit the record button at the beginning of the experiment. This resulted in a final sample of 108 participants (55 dental pain, 53 mortality salience). Participants were rewarded with money (€ 25) or partial course credit.

### Physiological Measures

We recorded continuous electrocardiography (ECG) and impedance cardiography (ICG) using a BIOPAC MP150 amplifier (Bipoac Systems Inc., Goleta, CA, United States). Respiration and electrodermal signals were recorded using a REFA 72 amplifier (TMSi, Oldenzaal, Netherlands). The REFA 72 amplifier was also used to record continuous electroencephalography (EEG) data. All information, manipulations, tasks, and questionnaires were run on a computer running Inquisit 4.0.9.0 (Millisecond). Physiological parameters were computed offline from the recorded data using ANSLAB (Blechert et al., 2016). Statistical analyses were done in R (R Foundation for Statistical Computing, 2016).

For the ECG, we used disposable Ag/AgCl pre-gelled spot electrodes in a lead II configuration (one placed at the right clavicle, one at the left lower ribcage; the ground electrode was placed at the right lower ribcage). Electrode sites were cleaned using with Nuprep skin preparation gel. The ECG signal was filtered with a 40 Hz low-pass filter, a 50 Hz notch filter, and a 0.5 Hz high pass filter, and resampled to 400 Hz. R waves were detected with an automatic algorithm implemented in ANSLAB. Results were manually checked and corrected if required. Average heart period (HP) was calculated for each stage of the experiment as the average interval between successive R-waves.

For measuring basal impedance (Z0) and change in impedance (dZ/dt), we used a Biopac NICO100C module. Two current-inducing disposable Ag/AgCl pre-gelled spot electrodes were placed at the neck and lower back. Two measuring disposable Ag/AgCl pre-gelled electrodes were put 5 cm below the upper and 5 cm above the lower current-inducing electrodes. Electrode sites were cleaned using with Nuprep skin preparation gel. The average distance between the two measuring electrodes was 48.28 cm (*SD* = 5.80 cm). The dZ/dt signal was filtered with a 50 Hz band-stop filter, and resampled to 1000 Hz before analysis. An automatic algorithm implemented in ANSLAB served to detect the B, Z, and X points in ensemble averages (type: median) of the dZ/dt signal. Results were manually checked and corrected if required. The Q-point which is needed to calculate PEP, was set at 20 ms before the R-wave. To calculate CO, we determined stroke volume (SV) by applying the Kubicek formula, where thoracic resistivity (rho) was assumed to be 135 Ohm ⋅ cm. From MAP and CO, we calculated total peripheral resistance (TPR; TPR = MAP/CO).

**FIGURE 1:**
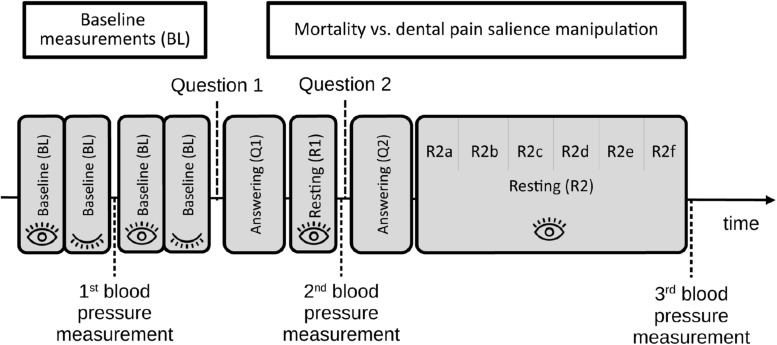
A schematic illustration of the experimental protocol.

Unlike the other physiological measures, systolic and diastolic BP (BPsys and BPdia) were not measured continuously, but at fixed points during the experimental protocol (see Figure 1) using a blood pressure monitor (Ecomed BU-90E, Medisana AG, Neuss, Germany). The first blood pressure measurement was taken in between the four baseline recordings, and served to calculate baseline TPR (BL; see Figure 1). The second blood pressure measurement was taken immediately after the resting interval following the first salience question, and served to calculate TPR during the responding and resting periods associated with question 1 (Q1 and R1; see Figure 1). The third blood pressure measurement was taken after the resting interval following question 2 and served to calculate TPR during the question 2 responding and resting periods (Q2 and R2; see Figure 1). Because we did not have different hypotheses for systolic or diastolic blood pressure, we calculated mean arterial pressure (MAP) based upon them: MAP = 1/3 ^∗^ BP_sys_ + 2/3 ^∗^ BP_dia_.

From a spectral analysis of individual inter-beat interval time-series, we calculated the high-frequency (HF-HRV) and low-frequency (LF-HRV) powers of heart rate variability as the natural logarithm of the power spectral density between 0.14 and 0.4 Hz, and 0.07 and 0.14 Hz, respectively. Because ECG artifacts can strongly bias estimates of heart rate variability (Berntson and Stowell, 1998), inter-beat interval time series were manually screened, and artifacts were detected and resolved before determining heart rate variability.

We measured respiratory activity using two inductive respiration belts placed around the thorax and the abdomen. In cases where more than one belt provided good data quality, respiratory rate was averaged across the two belts. A 0.033 Hz high-pass filter and a 1 Hz low-pass filter were applied, and the signal was resampled to 25 Hz. An automatic algorithm implemented in ANSLAB served to detect the onset and end of inspiration and expiration, respectively. Respiratory rate was calculated as the number of respiratory cycles per minute.

We recorded electrodermal activity using two Ag/AgCl electrodes placed on the middle phalanges of the index and middle finger of the non-dominant hand. The electrodes were coated with isotonic electrode paste and attached using velcro straps. The raw signal was resampled to 25 Hz prior to analysis. Qualifying onsets for spontaneous skin conductance fluctuations were automatically determined as local minima within the raw electrodermal activity signal. The minimum distance between two qualifying onsets was set to 1 s. Local maxima within a time window of 3.5–12 s following a qualifying point were counted as non-specific fluctuations when their amplitude exceeded 0.02 yS.

For each of the physiological parameters, averages were obtained for each of the experimental stages: baseline, during Question 1, following Question 1, during Question 2, and following Question 2. Because the interval following the second question was 6 min long, we subdivided it into six 1-min intervals to increase temporal resolution.

### Design

To manipulate mortality salience, we asked participants to answer two questions related to death or dental pain (between-subjects). The cover story for the procedure was that the questions were part of a so-called “projective life attitudes questionnaire,” an innovative personality test based on feelings and attitudes toward certain topics. Participants were told that their responses would be content-analyzed to draw conclusions about their personality, and that honest responses were therefore appreciated. In the mortality salience condition, the first question was “Please briefly describe the emotions that the thought of your own death arouses in you.” On average, participants spent 79.28 s (*SD* = 45.19 s) to answer the first question using a computer keyboard. In the dental pain condition, the first question was “Please briefly describe the emotions that the thought of a painful dental procedure arouses in you.” The average time spent on the first dental pain question was 81.29 s (*SD* = 40.01 s).

To determine whether the manipulation worked as intended, we used the tm package (Feinerer and Hornik, 2018) to determine the 30 most frequently occurring words in participants’ written answers in the mortality and dental pain salience groups. Punctuation, numbers, white spaces, and stop words were removed, and all words were converted to lower case.

We assessed several kinds of physiological activation data before, during and after participants answered the salience questions (see section “Procedure” for details). Figure 1 shows a schematic description of the experimental protocol.

### Procedure

Upon their arrival in the lab, participants were briefed about the study protocol, including the tasks and measurements involved. After providing written informed consent, they were fitted with sensors and seated in front of a computer screen on which the instructions and questions were presented.

To obtain baseline levels of physiological activation, data during four subsequent 1-min periods (two with eyes open, two with eyes closed) were recorded prior to the manipulation of mortality salience or dental pain. Blood pressure was measured between the second and third of these baseline recordings. After the first question, we measured blood pressure. Then, a 1-min resting interval with eyes open followed. This resting interval between the questions served to create a sampling period free of movement artifacts resulting from typing. Movement was a concern, especially with our measure of electrodermal activity, which was measured from the index and middle fingers. The second question in the mortality salience condition was “Jot down, as specifically as you can, what you think will happen to you as you physically die and once you are physically dead” (time taken to respond: *M* = 78.53 s, *SD* = 37.69 s). The second question in the dental pain condition was “Jot down, as specifically as you can, what you think will happen to you when you are at the dentist and undergo a painful dental procedure” (time taken to respond: *M* = 79.53 s, *SD* = 39.95 s). Time on task did not differ between the two experimental conditions [first question: *t*(106) = −0.24, *p* = 0.808; second question: *t*(106) = −0.13, *p* = 0.894]. After the second question, a 6-min resting interval followed. For data analysis, this resting interval was subdivided into six 1-min intervals to provide a more fine-grained temporal resolution and to make the data from the 6-min interval more comparable to that from the 1-min intervals. After the 6 min were over, we performed the third and final blood pressure measurement. Then, participants took part in another experiment in which they were asked to judge the parity of visually presented playing cards. After that, they answered several questionnaires assessing personal project zeal (McGregor et al., 2007), ethnocentrism (Bizumic et al., 2009), promotion-prevention regulatory focus strength (Sassenberg et al., 2012), mindfulness (Brown and Ryan, 2009), aggression (Buss and Perry, 1992), neuroticism (Rammstedt and John, 2007), need for cognitive closure (Schlink and Walther, 2007), anxiety and approach motivation (unpublished scales developed in-house), anxiety (Spielberger et al., 1970), self-efficacy (Schwarzer and Jerusalem, 1999), and self-esteem (Rosenberg, 1965). Since mortality salience effects have been reported to be moderated by variables such as the ones we assessed here (e.g., Harmon-Jones et al., 1997; Niemiec et al., 2010; Agroskin et al., 2016), it would have been straightforward to probe whether one or more of them moderated the effect of MS on arousal. However, we deliberately avoided probing these moderations due to the large number of number of statistical tests that would have resulted. Correcting for multiple comparisons would have reduced the expected number of false positives, but would have also increased the number of false negatives, making the moderator analysis strategy insensitive. At the end, sensors were removed and participants were debriefed, thanked, and rewarded.

### Statistical Analysis

#### Missing Data Management

From the 108 participants for whom data were available, 13.92% of data were missing due to excessive noise, temporary sensor malfunction, or loose contacts. To avoid statistical power fall-offs related to listwise deletion, as well as the emergence of different subsets of participants for different signals associated with pairwise deletion, we used multiple imputation (Graham, 2009) as implemented in the MICE package for R (van Buuren, 2018). We imputed five datasets, each based on five iterations, using the predictive mean matching method. We ran each statistical model based on one of the five imputed datasets, and subsequently pooled the estimates.

#### Hypothesis Testing Strategy

First, we used a series of *t*-tests to investigate how each of the nine physiological parameters differed from baseline during each of the nine post-baseline measurement periods under investigation, regardless of whether participants received the mortality or dental pain questions. Blood pressure was only measured twice post-baseline, thus providing two instead of nine values per participant. In a second set of linear regression models, we used another set of *t*-tests to investigate how changes in physiological parameters relative to baseline differed between the mortality and the dental pain salience conditions for each of the physiological parameters and measurement periods. To correct for alpha inflation caused by the large number of statistical tests (*n* = 148), we corrected the resulting *p*-values for multiple comparisons using false discovery rate (FDR), as implemented in the fdrtool package (Version 1.2.15, Klaus and Strimmer, 2015). Supplementary Table 1 shows both uncorrected and corrected *p*-values.

In addition to the significance testing approach described above, we analyzed Bayes factors for each of the 148 comparisons. The main goal was to tell the meaning of non-significant results – whether they constitute evidence for no effect, or reflect data insensitivity, which is not possible in conventional statistical testing (Dienes, 2014). We calculated Bayes factors from the *t* statistics using the BayesFactor package for R (Morey et al., 2018) with the scale factor set to 1. We used a Bayesian framework (Rouder et al., 2009) to address this problem and to determine whether the data actually provide evidence for the null hypothesis or not. In a Bayesian framework, three conclusions are possible with regard to the null and alternative hypotheses: First, there may be sufficient evidence for the alternative over the null (MS leads to more arousal than DPS). Second, there may be sufficient evidence to favor the null over the alternative (there is no difference between MS and DPS). Third, the data do not provide sufficient evidence to distinguish the hypotheses, and more data are needed to do so. The Bayes factor (*B*) is the ratio of the degree of evidence for one hypothesis or model (for example, the alternative hypothesis) over the degree of evidence for another hypothesis or model (for example, the null hypothesis). If that ratio is greater than 1, the alternative hypothesis received more support than the null hypothesis. An established rule is that *B*s greater than 3 represent moderate evidence for the alternative over the null hypothesis, and that *B*s smaller than 1/3 represent moderate evidence for the null over the alternative hypothesis. A B of 3 roughly corresponds to *p* < 0.05 in conventional statistical testing (Dienes, 2014). Throughout this paper, we will refer to effects for which we obtained Bayes factors below 1/3 as evidence for no effect, and effects for which the Bayes factor was between 1/3 and 3 as inconclusive.

#### Sensitivity and Agreement of the Conventional and Bayesian Statistical Analyses

The uncorrected conventional statistical analysis yielded significant values in 15.54% of the tests conducted. FDR correction decreased this number to 10.14%. The Bayesian analysis supported the alternative hypothesis in 12.16% of comparisons, suggesting that its sensitivity was in between the corrected and uncorrected conventional statistical analyses. There were three cases in which the significance testing and the Bayes factor approaches disagreed on whether an effect was significant or represented evidence for an effect, respectively (see Table 2). However, these differences were irrelevant to the main conclusions of the study.

#### Sample Size, Statistical Power, and Bayesian Prior Considerations

It is unclear what effect size to expect regarding physiological activation in the mortality salience paradigm, or when comparing mortality salience with dental pain salience, because there are few precursor studies. Moreover, the precursor studies relied on small sets of physiological parameters and time intervals, which likely led to over- or underestimation of the true effect size. In addition, to our knowledge, our study is the first to investigate respiration rate, cardiac output, total peripheral resistance, and blood pressure in the mortality salience paradigm. In light of these considerations, we refrained from using effect sizes from existing studies to determine the required sample size.

A *post hoc* sensitivity analysis of the present study revealed that given the sample size, an α error probability of 0.05, and a power (1–β error probability) of 0.80, the required effect size in order to get a significant result was *dz* = 0.27 for the within-subjects comparison, and *d* = 0.54 for the between-subjects comparison, mirroring the fact that within-subject tests are usually more sensitive than between-subject tests. It also suggests that the study was able to detect small within-subject effects, and medium between-subject effects sensu Cohen (1988) with a statistical power of 0.80. The meta-analytically determined between-subject effect of MS versus various control conditions on non-physiological outcomes has been found *r* = 0.35 (Burke et al., 2010), which also represents a medium-sized effect sensu Cohen (1988). This suggests that the sample size of the present study is adequate.

The difficulty associated with determining reasonable expectations regarding effect size also led us to choose a JZS prior (Rouder et al., 2009; Morey and Rouder, 2011) for the Bayes factor analysis. The JZS prior minimizes assumptions about the range of effect size and is, in this sense, an objective prior.

## Results

### Manipulation Check

We determined the 30 most frequently occurring words in participants’ written responses to the manipulation using a text mining algorithm. Participants in the mortality salience group made regular use of death-related words (e.g., death, body, soul, grief, belief, bury, or ground), and participants in ithe dental pain group regularly used dental pain-related words (e.g., pain, treatment, dentist, and quickly), but not vice versa (see Table 1). In addition, the words “I” and “anxiety” were the most frequently used word in both conditions. This suggests that the manipulation was successful, at least in the sense that participants had the right topics in mind.

**TABLE 1:** A list of the 30 most frequently used words occuring in participants written narratives, separately for the mortality salience and dental pain groups.

**Mortality salience condition**			**Dental pain condition**		
**German**	**English**	***n***	**German**	**English**	***n***
angst	anxiety	28	angst	anxiety	43
ich	I	27	ich	I	40
tod	death	24	dass	that	29
leben	life	23	schmerzen	pain	20
körper	body	22	versuche	try	17
mehr	more	16	behandlung	treatment	16
passiert	happens	16	schnell	quickly	11
dass	that	15	schmerz	pain	11
seele	soul	14	vorbei	over	10
trauer	grief	13	zahnarzt	dentist	8
danach	after	10	hoffe	hope	7
freunde	friends	10	vermutlich	probably	6
mein	my	9	nervös	nervous	6
glaube	belief	9	unruhig	restless	6
familie	family	7	davor	prior to that	6
gedanken	thoughts	7	würde	would	6
vielleicht	maybe	7	gefühl	feeling	6
einfach	simply	7	einfach	simply	6
begraben	bury	7	bringen	bring	6
erde	ground	7	denke	think	6
kommt	comes	6	ruhig	calm	6
denke	think	6	abzulenken	distract	6
für	for	6	denken	think	6
nichts	nothing	6	gedanken	thoughts	5
bzw	respectively	5	bald	soon	5
weiß	know	5	unangenehm	unpleasant	5
ruhe	calm	5	mache	do	4
der	the	5	unbehagen	unease	4
art	kind	5	unwohlsein	discomfort	4
verbrannt	burnt	5	fühle	feel	4

*Words are sorted by number of occurrences.*

### Physiological Activation in the Mortality Salience Paradigm

**FIGURE 2:**
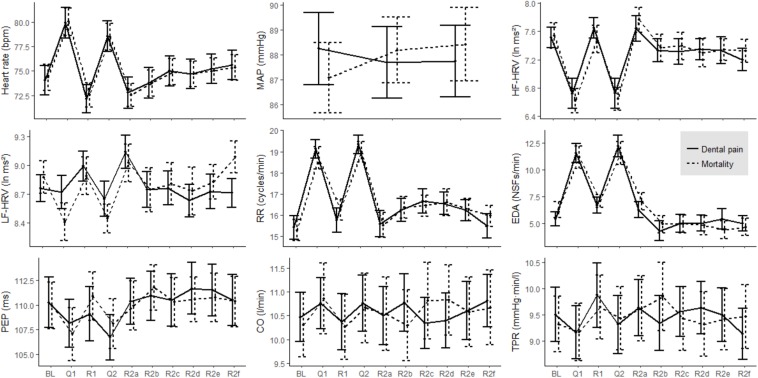
Time courses of physiological parameters as a function of segment (BL, baseline; Q1, first question; R1, 1-min resting interval following first question; Q2, second question; R2, 6-min resting interval following second question) and experimental condition (solid line – dental pain salience, dotted line – mortality salience). Error bars indicate standard errors of the mean. Note the spikes in heart rate, HF-HRV, RR, and EDA that coincide with the question periods. These indicators also exhibit delayed physiological activation in the resting interval following Question 2. Impedance cardiographic indicators (PEP, CO, and TPR) exhibited no reactivity to the MS manipulation. The mortality salience and dental pain conditions did not differ on any of the physiological measures in any of the segments.

First, we assessed how the physiological indicators differed from baseline during each of the nine post-baseline measurement periods under investigation, regardless of whether participants received mortality or dental pain questions. The results are illustrated in Figure 2. Table 2 provides statistical information.

**TABLE 2:** Mean differences, standard errors, and *B* values related to the comparison of each segment with baseline (upper half) and the mortality salience > dental pain salience comparisons (lower half).

	**Segment**								
	**Ql**	**Rl**	**Q2**	**R2a**	**R2b**	**R2c**	**R2d**	**R2e**	**R2f**
**Segments > Baseline**									
**Mean differences**									
HR (bpm)	3.57	–1.18	2.77	–0.93	–0.33	0.45	0.30	0.63	0.89
MAP (mmHg)	–	0.27	–	–	–	–	–	–	0.41
In HF-HRV (ms^2^)	–0.79	0.08	–0.88	0.13	–0.20	–0.16	–0.21	–0.22	–0.25
In HF-HRV (ms^2^)	–0.20	0.18	–0.26	0.25	–0.05	–0.03	–0.09	–0.04	0.09
RR (cycles/min)	3.68	0.78	3.70	0.18	1.07	1.50	1.34	0.95	0.01
NSFR (n/min)	5.84	0.39	6.33	0.48	–1.18	–0.95	–0.72	–0.27	–1.07
PEP (ms)	–3.10	–0.68	–3.43	–0.71	1.54	0.01	1.39	1.36	0.02
CO (l/min)	0.37	0.89	0.69	0.03	–0.22	–0.09	–0.10	–0.07	0.13
TPR (mmHg min/l)	–0.24	0.35	–0.04	0.22	0.18	0.08	0.06	0.05	–0.11
**Standard errors**									
HR (bpm)	–0.28	–0.21	–0.30	–0.24	–0.30	–0.29	–0.26	–0.27	–0.27
MAP (mmHg)	–	0.56	–	–	–	–	–	–	0.44
In HF-HRV (ms^2^)	0.12	0.07	0.14	0.08	0.07	0.07	0.07	0.08	0.08
In HF-HRV (ms^2^)	0.13	0.08	0.13	0.09	0.08	0.09	0.10	0.09	0.09
RR (cycles/min)	0.77	0.58	0.63	0.57	0.65	0.46	0.51	0.57	0.54
NSFR (n/min)	0.82	0.58	1.39	1.00	0.85	0.85	0.91	1.00	0.80
PEP (ms)	2.27	2.26	2.48	1.78	1.86	2.62	2.10	2.18	1.74
CO (l/min)	0.47	1.20	0.57	0.41	0.53	0.47	0.46	0.43	0.51
TPR (mmHg min/l)	0.17	0.17	0.20	0.20	0.18	0.14	0.16	0.16	0.17
***B* values**									
HR (bpm)	>1000^a^	>1000^a^	>1000^a^	106.23^a^	0.26_n_	0.46	0.28_n_	1.98	24.15^a^
MAP (mmHg)	–	0.17_n_	–	–	–	–	–	–	0.22_n_
In HF-HRV (ms^2^)	>1000^a^	0.31_n_	>1000^a^	0.54	5.07^a^	2.31	11.65^a^	3.10^a^	19.31^a^
In HF-HRV (ms^2^)	0.45	1.52	1.04	6.01^a^	0.18_n_	0.16_n_	0.21_n_	0.16_n_	0.23_n_
RR (cycles/min)	>1000^a^	0.34	>1000^a^	0.16_n_	0.52	17.89^a^	3.58^a^	0.54	0.15_n_
NSFR (n/min)	>1000^a^	0.18_n_	>1000^a^	0.17_n_	0.36	0.27_n_	0.20_n_	0.15_n_	0.35
PEP (ms)	0.36	0.15_n_	0.37	0.16_n_	0.20_n_	0.15_n_	0.18_n_	0.18_n_	0.15_n_
CO (l/min)	0.20_n_	0.19_n_	0.30_n_	0.15_n_	0.16_n_	0.15_n_	0.15_n_	0.15_n_	0.15_n_
TPR (mmHg min/l)	0.40	1.12	0.15_n_	0.27_n_	0.25_n_	0.17_n_	0.16_n_	0.15_n_	0.18_n_
**Mortality salience > Dental pain salience**									
**Mean differences**									
HR (bpm)	–0.06	0.01	–0.24	–0.18	–0.25	–0.46	–0.39	–0.53	–0.39
MAP (mmHg)	–	1.44	–	–	–	–	–	–	1.73
In HF-HRV (ms^2^)	0.24	–0.07	0.05	0.05	–0.02	0.15	0.02	0.09	0.19
In HF-HRV (ms^2^)	–0.24	–0.13	–0.29	–0.21	–0.10	–0.02	0.07	0.09	0.27
RR (cycles/min)	–0.65	0.70	–0.30	0.05	0.16	0.08	0.45	–0.10	0.36
NSFR (n/min)	–0.15	–0.64	–0.77	–0.38	0.11	–0.81	–0.54	–0.82	–1.47
PEP (ms)	–1.82	0.60	–1.15	–1.37	–0.05	–2.60	–3.38	–3.12	–0.47
CO (l/min)	0.91	2.08	0.90	1.16	0.62	1.17	0.85	0.72	0.85
TPR (mmHg min/l)	0.05	0.04	0.24	0.12	0.71	0.01	–0.16	0.04	0.42
**Standard errors**									
HR (bpm)	–0.53	–0.43	–0.57	–0.54	–0.56	–0.54	–0.53	–0.57	–0.51
MAP (mmHg)	−	1.03	−	−	−	−	−	−	0.85
In HF-HRV (ms^2^)	0.25	0.13	0.25	0.14	0.14	0.14	0.13	0.16	0.15
In HF-HRV (ms^2^)	0.24	0.16	0.22	0.17	0.17	0.17	0.19	0.18	0.17
RR (cycles/min)	0.74	0.83	0.69	1.06	1.51	0.75	1.05	0.94	0.94
NSFR (n/min)	2.11	1.20	1.51	1.29	1.18	1.29	1.57	1.66	1.49
PEP (ms)	3.87	3.80	3.32	3.45	3.42	4.74	3.25	3.90	3.29
CO (l/min)	0.86	2.27	0.91	0.98	1.08	0.90	0.74	0.78	0.75
TPR (mmHg min/l)	0.34	0.34	0.41	0.40	0.35	0.30	0.32	0.33	0.35
***B* values**									
HR (bpm)	0.15_n_	0.15_n_	0.16_n_	0.16_n_	0.16_n_	0.21_n_	0.19_n_	0.22_n_	0.20_n_
MAP (mmHg)	–	0.37	–	–	–	–	–	–	1.01
In HF-HRV (ms^2^)	0.23_n_	0.17_n_	0.15_n_	0.16_n_	0.15_n_	0.26_n_	0.15_n_	0.17_n_	0.34
In HF-HRV (ms^2^)	0.24_n_	0.21_n_	0.33_n_	0.30_n_	0.17_n_	0.15_n_	0.16_n_	0.17_n_	0.53
RR (cycles/min)	0.21_n_	0.21_n_	0.16_n_	0.15_n_	0.15_n_	0.15_n_	0.16_n_	0.15_n_	0.16_n_
NSFR (n/min)	0.15_n_	0.17_n_	0.17_n_	0.15_n_	0.15_n_	0.18_n_	0.16_n_	0.17_n_	0.23_n_
PEP (ms)	0.16_n_	0.15_n_	0.16_n_	0.16_n_	0.15_n_	0.17_n_	0.25_n_	0.20_n_	0.15_n_
CO (l/min)	0.25_n_	0.22_n_	0.24_n_	0.29_n_	0.17_n_	0.33_n_	0.28_n_	0.22_n_	0.27_n_
TPR (mmHg-min/l)	0.15_n_	0.15_n_	0.17_n_	0.15_n_	0.96	0.15_n_	0.17_n_	0.15_n_	0.29_n_

*HR, heart rate; MAP, mean arterial pressure; HF-HRV, high-frequency heart rate variability; LF-HRV, low-frequency heart rate variability; RR, respiration rate; NSFR, non-specific fluctuation rate; PEP, pre-ejection period; CO, cardiac output; TPR, total peripheral resistance; ^*a*^Bayes factor supports alternative over null hypothesis; _*n*_Bayes factor supports null over alternative hypothesis.*

While responding to the questions, heart rate (HR), respiration rate, and electrodermal activity increased, whereas HF-HRV decreased, indicating higher arousal than at baseline. Cardiac output remained unchanged. The results were inconclusive with regard to LF-HRV, PEP, and TPR (no effect for TPR during Question 2).

In the resting interval following the first question, heart rate sank below the baseline level, indicating lower arousal than at baseline. MAP, electrodermal activity, PEP, and CO did not differ from baseline. The results were again inconclusive with regard to TPR. The first minute following Question 2 was also marked by heart rates lower than baseline, indicating reduced arousal. There was an increase in LF-HRV. HF-HRV results were inconclusive. We obtained evidence for null findings for RR, NSFR, PEP, CO, and TPR.

During much of the 6-min resting interval, heart rate was at baseline. Toward the end, HR increased, and eventually exceeded its baseline level in the last minute. A similar, even more pronounced picture emerged for HF-HRV. Respiration rate increased above baseline during minutes three and four, but was not different from baseline levels during the first and last minute. NSFR scores were not different from baseline or inconclusive throughout the 6-min period. PEP, CO, and TPR did not differ from baseline throughout the entire 6-min interval.

### Physiological Activation: Differences Between Mortality Salience and Dental Pain

No significant differences were observed in any of the physiological measures as a function of whether participants received the mortality salience or the dental pain salience questions. These results mostly represented evidence for no effect, except for six cases where the data were insensitive: mean arterial pressure (MAP), HF-HRV during Question 2, TPR during Question 2, and HF- and LF-HRV during the sixth minute following Question 2.

## Discussion

This study’s goal was to investigate physiological arousal in the mortality salience paradigm and to test whether MS creates more physiological arousal than dental pain salience (DPS), a common control condition for MS. Overcoming some of the limitations of previous physiological studies on MS, we employed a broad array of measures, a generous number of measurement intervals, and ICG to investigate challenge versus threat cardiovascular patterns. Answering the MS and DPS questions both led to short-lived as well as delayed changes in physiological arousal. Importantly, Bayes Factor analyses revealed evidence for no difference between MS and DPS. These results cast doubt on the idea that shifts toward worldview-defensive and self-esteem-defense in response to mortality salience occur because mortality salience produces higher physiological activation than dental pain salience.

Responding to the questions clearly elicited arousal, as reflected by increases in heart rate, respiration rate, and electrodermal activity, and decreases in high-frequency heart rate variability (HF-HRV), which is inversely related to arousal (Grossman et al., 1991; Grossman and Kollai, 1993; Berntson et al., 1997). In the minute after participants answered the questions, none of these effects were apparent anymore. On the contrary, heart rate was even lower than at baseline. This post-question dip may have emerged because participants used automatic emotion regulation strategies (Mauss et al., 2007). In fact, automatic emotion regulation has been found to affect heart rate in anxiety-eliciting tasks (Williams et al., 2009).

Low-frequency heart rate variability (LF-HRV), which has previously been found to be increased while taking a virtual reality tour in a graveyard versus in a park (Chittaro et al., 2017), increased selectively during the first minute following the second question. This measure is difficult to interpret because it is influenced by multiple factors, including both sympathetic and parasympathetic outflows and baroreceptor activity (Malliani et al., 1994; Acharya et al., 2006; Goldstein et al., 2011). In the present study, LF-HRV did not exhibit the same question-related increases that we saw with heart rate, respiration rate, electrodermal activity, and high-frequency heart rate variability. This may indicate that LF-HRV may be a poor indicator of physiological arousal, and reflects different physiological processes that may nevertheless be relevant in the context of mortality salience.

We observed a delayed return of arousal across several indicators during the course of the 6-min resting interval following Question 2. Respiration rate was higher during the third and fourth minute than at baseline. HF-HRV was higher from the fourth to the sixth minute. Heart rate was elevated in the final minute. There are several ways to interpret this delayed increase in arousal. First, it could be the physiological reflection of a rebound effect that has been proposed to happen in the MS paradigm (Pyszczynski et al., 1999): When writing about their own death, people tend to suppress death-related thoughts. Thought suppression often fails (Wegner, 1994), and the thoughts to be suppressed tend to reemerge (Wegner and Smart, 1997). This re-emergence of death thoughts after some delay and distraction has been proposed to trigger worldview defense (Pyszczynski et al., 1999; Greenberg et al., 2000). Our delayed arousal findings suggest that in the mortality salience paradigm, physiological arousal may follow a similar trajectory to death-related thoughts: they are high while death is contemplated, drop, and rise again slowly. Because we also observed this pattern of delayed arousal in the dental pain condition, arousal is unlikely to explain why mortality salience typically leads to *more* worldview defense than dental pain salience. Alternatively, the delayed increase in arousal may occur simply because participants get bored and impatient while having to sit still for 6 min. A comparison with an affectively neutral control condition may help solve this issue: if reflecting on affectively neutral topics causes the same delayed increase in arousal, the affectively negative content of the mortality and dental pain questions cannot account for it. Interestingly, the delayed arousal increase was apparent in measures of heart rate and its variability, but not in other measures, such as skin conductance or respiratory rate. This illustrates the fact that different indicators of arousal can behave in different ways, underscoring the importance of considering multiple arousal indicators at the same time.

Reflecting on the mortality salience and dental pain questions produced no changes in cardiac output and total peripheral resistance. In the framework of the biopsychosocial model of arousal regulation (Blascovich and Tomaka, 1996; Blascovich, 2008), these measures can be used to indicate cardiovascular patterns of challenge and threat. However, according to that model, challenge and threat patterns emerge only in motivated performance situations which (i) involve goals that are important to the individual, and (ii) are active (i.e., require certain instrumental actions). First, the goal of writing about aversive topics may not be personally relevant enough in order to create a motivated performance situation. Second, although the task was “active” in the sense that it required typing, it might not qualify as an active task in the framework of the biopsychosocial model. This is because the content of the written responses did not determine whether participants could successfully complete their participation in the experiment. Our observation that mortality salience did not reduce PEP, an indicator of ventricular contractility, supports the idea that participants did not experience the task as a motivated performance situation (Seery et al., 2009; Behnke and Kaczmarek, 2018). This does not rule out that thoughts of mortality influence cardiovascular patterns of threat and challenge, but a more personally relevant and active situational context (such as anticipating giving a speech in front of an audience) may be required for these effects to become apparent.

We found no evidence for differences between MS and DPS. How can we reconcile this finding with results from misattribution studies which indicate that arousal plays a role in mortality salience effects (Greenberg et al., 2003) and other threats (Ben-Zeev et al., 2005; Proulx and Heine, 2008; Kay et al., 2010; Nash et al., 2011; Greenaway et al., 2015; Webber et al., 2015)? First, the subjective aspect of arousal (i.e., the self-reported strength of arousal or experienced emotional intensity), but not physiological arousal itself, may be important for MS effects to occur. Indeed, affect has been shown to mediate some MS effects (Echebarria-Echabe, 2013; Echebarria Echabe and Perez, 2016). This may also explain why “cognitive” manipulations of arousal eliminate defense, although mortality salience does not actually lead to greater physiological arousal. Work on response system coherence in emotions suggests that the relationship between physiological arousal and emotion experience is minor, and that the two levels of analysis are by no means interchangable (see e.g., Mauss et al., 2005). Therefore, even if MS would elicit more arousal than a negative control topic, affective responses (and mediation via them) may be relatively independent of peripheral physiological changes.

Secondly, the interaction of physiological and self-reported arousal may be the key. MS may lead to a state in which people think they are less aroused than they actually are, which could represent a case of “residual” arousal. According to excitation transfer theory (Zillmann et al., 1972; Zillmann et al., 1974), individuals often do not sense elevated physiological arousal after an arousal-inducing event (even after physical activity, which is emotionally neutral). Divergences between actual and perceived arousal have been found to intensify emotional reactions, highlighting a plausible psychophysiological mechanism underlying the more polarized evaluations of worldview-relevant targets following MS. Future studies should investigate the plausibility of that explanation. A fourth possibility is that some form of arousal is necessary but not sufficient for the emergence of worldview defense following MS. In other words, it may act as a moderator rather than a mediator. One study has shown that reappraisal and arousal misattribution procedures, which are aimed at reducing arousal, are able to prevent the emergence of death-thought accessibility following threat manipulations (Webber et al., 2015). Hence, arousal misattribution may paradoxically exert its influence via death-thought accessibility instead of arousal or affect. Future studies should pay attention to these complex possibilities.

A fifth possibility is that the kind of arousal produced by MS is neither observable in peripheral physiological activation nor conscious reports of arousal, and is better characterized as a state of “background alarm” or “unconscious vigilance” (Holbrook et al., 2011). The latter study has demonstrated that participants primed with mortality salience exhibited exaggerated ratings of valenced yet worldview-unrelated targets, and that exaggerated responses to worldview-related targets can be elicited by subliminal presentations of affective yet death-unrelated words or images.

Another possibility is that arousal really is not important to mortality salience effects, and the misattribution results can be explained in alternative ways. Indeed, several alternative explanations for arousal misattribution effects have been proposed (for a review, see Reisenzein, 1983). The attention-diversion hypothesis (Loftis and Ross, 1974) states that the experimental group (the one that receives an ostensibly arousing treatment) perceives the arousal caused by a subsequent emotion-eliciting event to be more surprising, interesting, or alarming than the control group (which receives treatments that are ostensibly calming or arousal-unrelated) does. This might draw attention away from the emotional, worldview-related stimuli presented later on, thereby dampening reactivity to them. As per the preparatory information hypothesis (Loftis and Ross, 1974; Calvert-Boyanowski and Leventhal, 1975), receiving ostensibly arousing treatments generates plausible expectations of arousal before being confronted with a threat. This reduces ambiguity and uncertainty rather than arousal itself, which, in turn, explains the attenuated reactions. To our knowledge, these alternative explanations have not been investigated to date, but should be considered in the quest to illuminate the mediating mechanisms of mortality salience and other threats.

Finally, it is possible that physiological dynamics operating in between mortality threat and defensive responding are qualified by individual factors reflecting vulnerability or responsivity. Such moderated mediations have been reported in the context of pain (Wolff et al., 2008) and stress (Peters et al., 2003).

### Limitations

Perhaps the biggest limitation is that we did not assess any of the other mediators that have been proposed and demonstrated to mediate MS effects, including death-thought accessibility, self-esteem, affect, or conscious aspects of arousal, in addition to physiological activation. It would have been interesting to see how physiological measures relates to these other measures following the mortality salience manipulation. Participants may have experienced one salience induction as more arousing or aversive than the other, which in turn may have masked between-group differences in physiological arousal. Another limitation is that we relied on single-shot measurements of blood pressure instead of averaging across repeated measurements to account for the relative unreliability of ambulatory blood pressure devices (Shapiro et al., 1996). This problem also affects the measurement of total peripheral resistance, which we calculated based on blood pressure.

## Conclusion

Over the decades, the mortality salience paradigm has proven to be a useful tool to characterize and investigate the defensive efforts that humans employ in their struggle with the awareness of mortality. Misattribution of arousal may represent a “missing link” between threats such as mortality awareness and related coping efforts. Measuring physiological arousal constitutes an attractive way to test this idea, because biosignals can be measured objectively and reliably. Using a comprehensive setup, this study showed that a mortality salience manipulation does indeed lead to changes in arousal. These changes, however, are indistinguishable from those elicited by dental pain salience, a common control condition. Future studies should explore whether the interaction between different forms of arousal (e.g., phsyiological and self-reported) shapes defensive responses to mortality salience. We hope that our results will encourage and inspire new and more complex ideas regarding arousal and its role in the mortality salience paradigm, and ultimately contribute to identifying the mediating process(es) of mortality-salience-induced defense.

## Data Availability

The datasets generated for this study are available on request to the corresponding author.

## Ethics Statement

This study was carried out in accordance with the recommendations of the University of Salzburg’s ethics committee. All subjects gave written informed consent in accordance with the Declaration of Helsinki. The protocol was approved by the University of Salzburg’s ethics committee.

## Author Contributions

JK conceived and designed the experiments, acquired and analyzed the data, and wrote the manuscript. EJ contributed to the manuscript and helped to shape the project in all its stages.

## Conflict of Interest Statement

The authors declare that the research was conducted in the absence of any commercial or financial relationships that could be construed as a potential conflict of interest.

## Abbreviations

CO: cardiac output
EDA: electrodermal activity
HF-HRV: high-frequency heart rate variability
LF-HRV: low-frequency heart rate variability
MAP: mean arterial pressure
NSF: non-specific fluctuations
PEP: pre-ejection period
RR: respiration rate
TPR: total peripheral resistance.
